# Benign and malignant focal liver lesions displaying rim arterial phase hyperenhancement on CT and MRI

**DOI:** 10.1186/s13244-024-01756-y

**Published:** 2024-07-18

**Authors:** Francesco Matteini, Roberto Cannella, Lorenzo Garzelli, Marco Dioguardi Burgio, Riccardo Sartoris, Giuseppe Brancatelli, Valérie Vilgrain, Maxime Ronot, Federica Vernuccio

**Affiliations:** 1 Department of Biomedicine, Neuroscience and Advanced Diagnostics (Bi.N.D.), University Hospital of Palermo, Palermo, Italy; 2 Department of Health Promotion, Mother and Child Care, Internal Medicine and Medical Specialties (PROMISE), University of Palermo, Palermo, Italy; 3 Department of Radiology, Hôpital Beaujon, AP-HP.Nord, Paris, France; 4 Université Paris Cité, INSERM U1149, “Centre de Recherche sur l’Inflammation”; CRI, Paris, France

**Keywords:** Liver neoplasm, Hepatocellular carcinoma, Magnetic resonance imaging, Computed tomography, Contrast media

## Abstract

**Abstract:**

Rim arterial phase hyperenhancement is an imaging feature commonly encountered on contrast-enhanced CT and MRI in focal liver lesions. Rim arterial phase hyperenhancement is a subtype of arterial phase hyperenhancement mainly present at the periphery of lesions on the arterial phase. It is caused by a relative arterialization of the periphery compared with the center of the lesion and needs to be differentiated from other patterns of peripheral enhancement, including the peripheral discontinuous nodular enhancement and the corona enhancement. Rim arterial phase hyperenhancement may be a typical or an atypical imaging presentation of many benign and malignant focal liver lesions, challenging the radiologists during imaging interpretation. Benign focal liver lesions that may show rim arterial phase hyperenhancement may have a vascular, infectious, or inflammatory origin. Malignant focal liver lesions displaying rim arterial phase hyperenhancement may have a vascular, hepatocellular, biliary, lymphoid, or secondary origin. The differences in imaging characteristics on contrast-enhanced CT may be subtle, and a multiparametric approach on MRI may be helpful to narrow the list of differentials. This article aims to review the broad spectrum of focal liver lesions that may show rim arterial phase hyperenhancement, using an approach based on the benign and malignant nature of lesions and their histologic origin.

**Critical relevance statement:**

Rim arterial phase hyperenhancement may be an imaging feature encountered in benign and malignant focal liver lesions and the diagnostic algorithm approach provided in this educational review may guide toward the final diagnosis.

**Key Points:**

- Several focal liver lesions may demonstrate rim arterial phase hyperenhancement.
- Rim arterial phase hyperenhancement may occur in vascular, inflammatory, and neoplastic lesions.
- Rim arterial phase hyperenhancement may challenge radiologists during image interpretation.

**Graphical Abstract:**

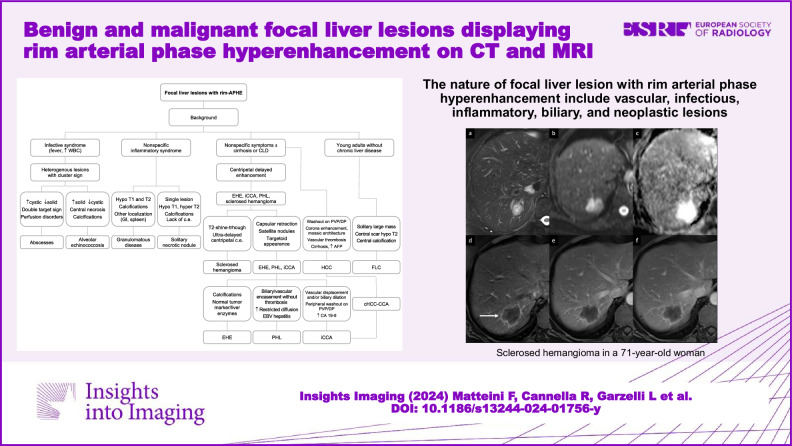

## Introduction

Peripheral arterial enhancement of focal liver lesions is an imaging feature that encompasses different patterns occurring in the arterial phase (AP) on contrast-enhanced CT and/or MRI, including the presence of peripheral nodular discontinuous enhancement, rim arterial phase hyperenhancement (rim APHE), and corona enhancement. Rim APHE is defined as the presence of hyperenhancement of the peripheral portions of a lesion during the AP due to relative arterialization of the periphery compared to the center [1]. Peripheral discontinuous nodular enhancement is a temporal enhancement pattern that progresses centripetally and parallels the blood pool from the AP to the portal venous (PVP) and delayed phases (DP). It is typically encountered in cavernous hemangiomas [1]. Corona enhancement is characterized by a perilesional enhancement with variable thickness and flame-shaped borders in late AP or early PVP. The enhancement is contiguous to the lesion and surrounds all or part of the lesion. Corona enhancement is part of the Liver Imaging Reporting and Data System version 2018 (LI-RADSv2018) ancillary features favoring malignancy in general but not specific for hepatocellular carcinoma (HCC) in high-risk patients [1, 2].

**Fig. 1:**
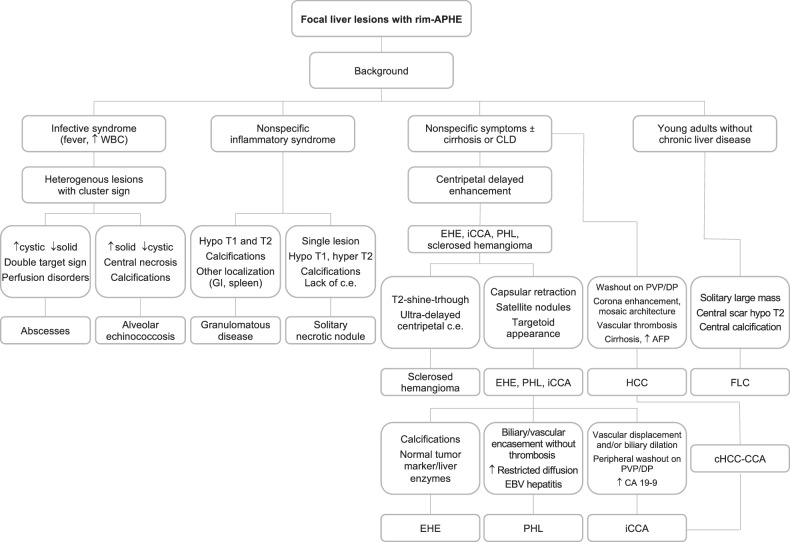
Proposed diagnostic algorithm flowchart for distinguishing between different focal liver lesions with rim peripheral enhancement. APHE, arterial phase hyperenhancement; CDL, chronic liver disease; EHE, epithelioid hemangioendothelioma; iCCA, intrahepatic cholangiocarcinoma; PHL, primary hepatic lymphoma; HCC, hepatocellular carcinoma; cHCC-CCA, combined hepatocellular-cholangiocarcinoma tumor; FLC, fibrolamellar hepatocellular carcinoma; EBV, Epstein–Barr virus; c.e., contrast enhancement

In the general population, rim APHE may be encountered in benign liver lesions with vascular, infectious, or inflammatory origin and malignant lesions, including both primary and secondary malignancies, with the most common being cholangiocarcinoma and metastases (Fig. 1). Benign and malignant focal liver lesions may display rim APHE as a typical imaging feature or an atypical presentation due to internal changes within the lesion. In patients at high risk for HCC (i.e., cirrhosis, chronic hepatitis B infection, or current/prior history of HCC), the presence of rim APHE is recognized as one of the main imaging features classifying an observation as LR-M category, i.e., definitely or probably malignant, but not specific for HCC [1, 2]. However, outside of the high-risk HCC population, benign entities may also exhibit rim APHE [3], and a multiparametric approach to MRI sequences—as well as the assessment of patient history and laboratory tests—may allow narrowing the list of differentials.

This review article aims to review the broad spectrum of focal liver lesions that may show rim APHE, using an approach based on the benign and malignant nature of lesions and their histologic origin.

## Benign

### Vascular

#### Sclerosed hemangioma

**Fig. 2:**
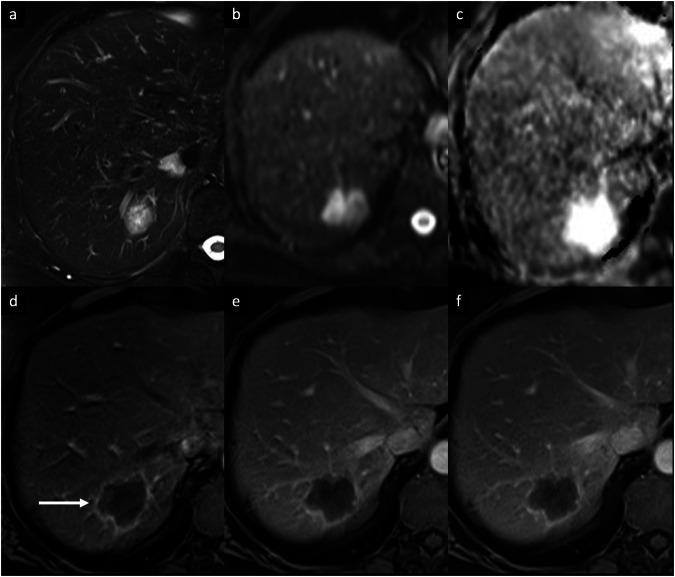
Sclerosed hemangioma in a 71-year-old woman who presented with abdominal pain and inflammatory syndrome. **a** Axial T2-weighted MRI shows a focal liver lesion in the right lobe with increased SI, with a hyperintense signal on diffusion-weighted imaging (**b**) but with high intensity on the ADC map (**c**). Extracellular contrast agent-enhanced MRI demonstrates a rim APHE (arrow) (**d**) that persisted and minimally increased on portal venous (**e**) and delayed phases (**f**). A biopsy of the lesion confirmed the diagnosis of sclerosed hemangioma

Sclerosed hemangiomas are rare, and they can be considered an end stage of hemangioma involution, characterized by various degenerative changes such as extensive fibrosis with subsequent hyalinization, marked narrowing or obliteration of the vascular spaces, and hemorrhage or sclerosis [4, 5]. Sclerosed hemangiomas are more commonly encountered in cirrhotic liver and may show over time capsular retraction, decrease in size, loss of previously seen regions of enhancement, or fibrotic changes [5, 6]. On contrast-enhanced CT, variable enhancement patterns have been reported. Sclerosed hemangiomas may show a rim APHE that persists on PVP (Fig. 2), with irregular regions of delayed enhancement within the lesions that manifested as areas of mild hyperattenuation compared with adjacent liver [7–9]. In addition, an arterioportal shunt in the AP that fades in the PVP may occur at the periphery of the lesion [8, 9]. On MRI, sclerosed hemangioma shows variable signal intensity (SI) on T2-weighted imaging (WI), particularly in larger lesions in which internal fibrotic septa show low or heterogeneous SI on T1-WI and high SI on T2-WI, oval-shaped contours, and liver capsular retraction [8, 9]; compared with cavernous hemangioma, the stroma of the sclerosed hemangioma contained abundance of collagenous tissue and elastic fibers around and between small sclerotic vessels [8, 9]. Tips for the radiological characterization include the presence of T2-shine-through on the apparent diffusion coefficient (ADC) map and the high SI on T2-WI, although usually slightly lower than typical hemangiomas (Fig. 2). In addition, challenging cases may benefit from ultra-DP acquisitions at 10 min after intravenous administration of extracellular contrast agents to observe the centripetal enhancement and contrast retention within the lesion [8–10]. On gadoxetate disodium-enhanced magnetic resonance imaging (Gd-EOB-MRI), sclerosed hemangioma appears hypointense compared to the background liver parenchyma during the transitional and hepatobiliary phases, with marginal hyperintensity in the peripheral area due to fibrous changes [7–9].

### Infectious

#### Abscess

**Fig. 3:**
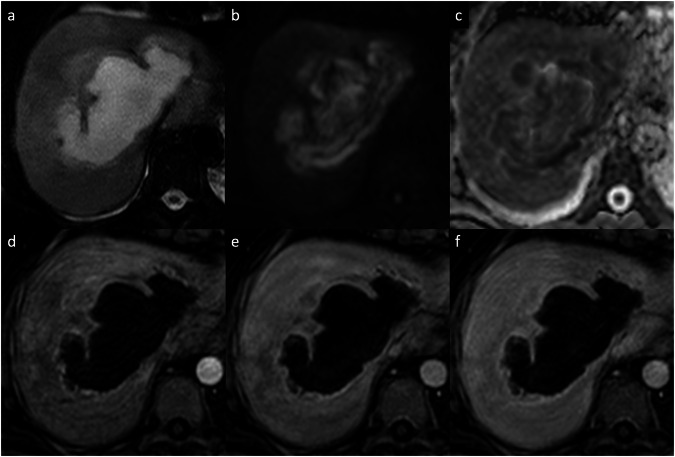
Amebic liver abscess in a 68-year-old man who presented with fever and right upper quadrant pain. Contrast-enhanced MRI using an extracellular contrast shows a large focal liver lesion, with (**a**) high SI on T2-weighted images, showing a “double target sign” with an iso- to hypointense inner layer and a hyperintense outer layer on T2-WI. The lesion shows (**b**) high SI on DWI (*b* = 800), (**c**) a peripheral low signal on the ADC map. **d** On the AP, the lesion demonstrates a thin-rim APHE, that persists (**e**) on the portal venous and (**f**) delayed phases

Abscesses can result from hematogenous dissemination of gastrointestinal infections via the portal vein or disseminated sepsis via the hepatic artery [11–13]. Bile infection, favored by duct obstruction from various etiologies, including stones, neoplasms, and strictures, is another frequent source of infection. Biliary stents and biliary-enteric anastomosis are also iatrogenic predisposing factors for pyogenic liver abscesses [11–13]. Hepatic infection by continuity, such as hepatic abscess from cholecystitis or direct introduction of bacteria into the liver parenchyma (during hepatic biopsy or surgery), and superinfection of pre-existing hepatic lesions, are other routes of liver abscesses [12, 13]. The clinical presentation includes fever, abdominal pain, nausea, leukocytosis, slightly elevated total bilirubin and aminotransferase levels, and hypoalbuminemia. On contrast-enhanced CT, pyogenic abscesses appear as single or multiple well-defined, hypoattenuating round lesions, ranging from a few millimeters (microabscesses) to several centimeters (macro abscesses), surrounded by a capsule [14]. The key imaging findings of large macro-abscesses are the layered-wall appearance, and they show an early inner wall rim APHE that persists in the DPs with a progressive delayed enhancement of the outer layer (“double target sign”); the entire lesion is often surrounded by segmental geographic or peripheral transient perfusion disorders, identified as regions with APHE that fade on PVP and DP [14–16]. The cluster sign is typical in abscesses of biliary origin and appears as multiple small hypoattenuating lesions with rim APHE that sometimes coalesce into larger lesions [14–16]. On MRI, abscesses show a central low SI on T1-WI and a central high SI on T2-WI, although the SI may vary depending on the proteinaceous content. The double target sign on MRI is represented by an iso- to hypointense inner layer and a hyperintense outer layer on T2-WI, with high SI of perilesional edema. Diffusion-weighted imaging (DWI) shows hyperintensity on high *b*-values and hypointensity on the ADC map [11–14]. The appearance of a pyogenic abscess on imaging is nearly indistinguishable from that of an amebic abscess. However, a solitary abscess is more likely to be amebic rather than pyogenic (Fig. 3) [15]. Although pyogenic abscesses usually appear to be fluid collections, they may also have a more solid appearance, mimicking primary or secondary hepatic tumors, such as intrahepatic cholangiocarcinoma (iCCA) or desmoplastic adenocarcinoma metastases. Areas of segmental or persistent rim enhancement at the periphery, perilesional edema surrounding organized abscesses, or associated findings of malignancy (capsular retraction, biliary duct dilatation, or lobar or segmental atrophy), are helpful additional imaging features that may help narrow the differential diagnosis between these entities [16, 17]. In some cases, aspiration/biopsy is needed to confirm the diagnosis [16, 17].

#### Alveolar echinococcosis

**Fig. 4:**
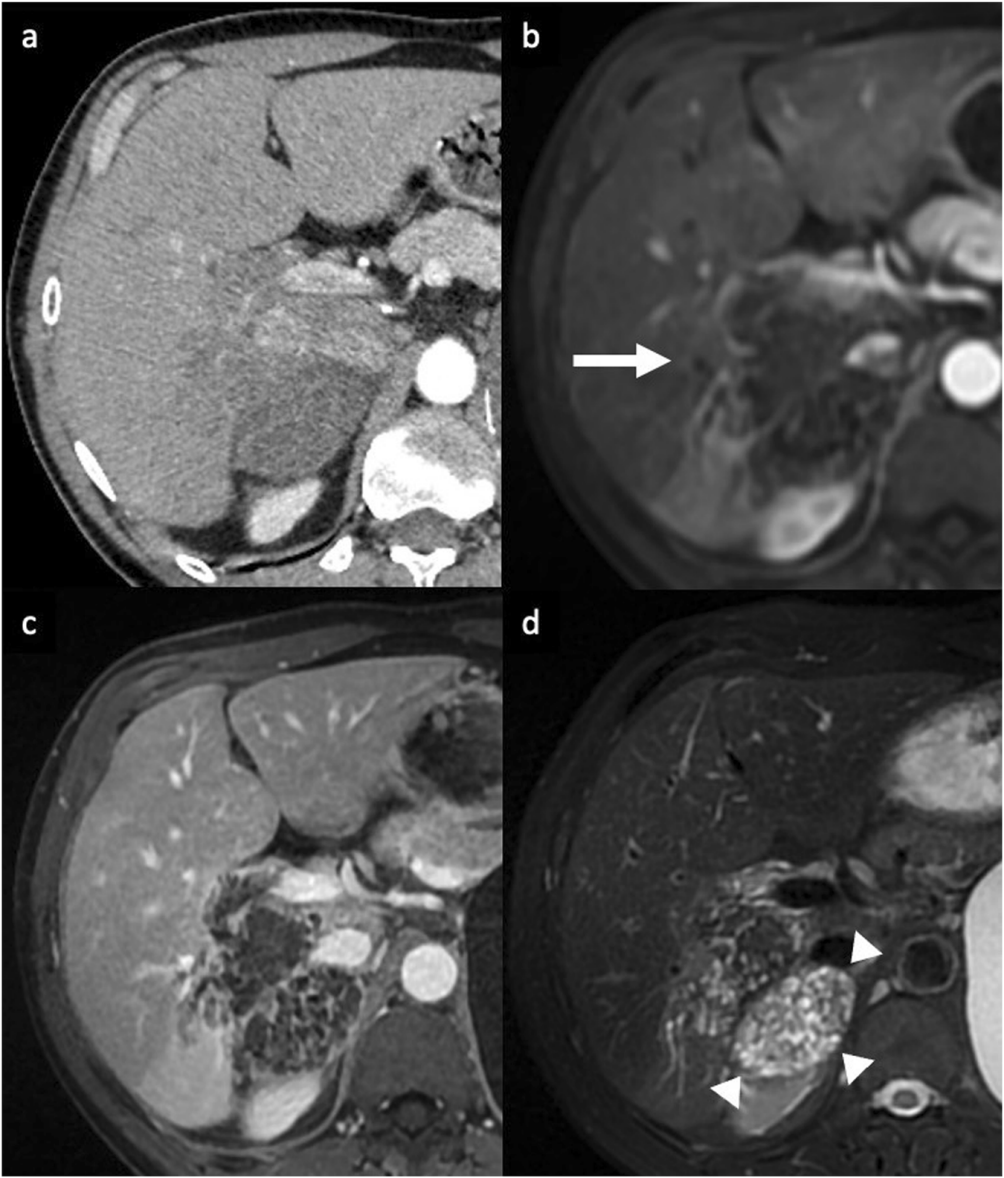
Alveolar echinococcosis in a 61-year-old man who presented with right upper quadrant pain and hypereosinophilia. **a** Contrast-enhanced CT shows a subcapsular exophytic liver lesion in the right hepatic lobe, containing hypoattenuating areas of necrosis and thin-rim APHE. On post-contrast MRI using an extracellular contrast agent, the lesion shows (**b**) a rim APHE (arrow) that persisted on (**c**) portal venous phases. **d** On axial T2-weighted MRI, the lesion appears slightly hyperintense, with the coalescence of multiple small cystic lesions (arrowheads) in a single larger cavity (“cluster sign”)

Echinococcus multilocularis is responsible for the rare alveolar echinococcosis. Alveolar echinococcosis occurs by either ingesting food or plants containing the eggs from the Echinococcus tapeworm or by direct contact with the definitive hosts, foxes [14, 18]. The liver is the most common site of infection (> 90% of patients). The lesion may be single or may appear as small, multilocular confluent heterogeneous cysts associated with solid components that demonstrate exogenous growth invading the adjacent hepatic parenchyma; a large cystic component is also frequently observed [18]. CT and MRI typically show multiple irregular, ill-defined lesions containing hypoattenuating areas of necrosis and active parasitic tissue hypoattenuating on CT and hyperintense on T2-WI on MRI, which show mild rim APHE or no contrast enhancement of the solid component [19]. The key imaging finding is the coalescence of multiple small cystic lesions in a single larger cavity (“cluster sign”) (Fig. 4). Hilar infiltration is common and results in dilatation of the intrahepatic bile ducts and invasion of the portal and hepatic veins, with subsequent atrophy of the affected liver segments due to hypoperfusion [18, 19].

### Inflammatory

#### Granulomatous diseases

Granulomatous hepatitis is an inflammatory liver disease associated with granuloma formation in the liver, and it is associated most commonly with sarcoidosis, tuberculosis, and histoplasmosis [20]. On contrast-enhanced imaging, these granulomatous diseases may occasionally present with multiple small hypoattenuating lesions showing subtle rim APHE. On MRI, the lesions are hypointense on T1-WI and hypo-to-isointense on T2-WI [20, 21]. Because of these relatively nonspecific findings, percutaneous liver biopsy is often performed for the definitive diagnosis.

#### Solitary necrotic nodule

**Fig. 5:**
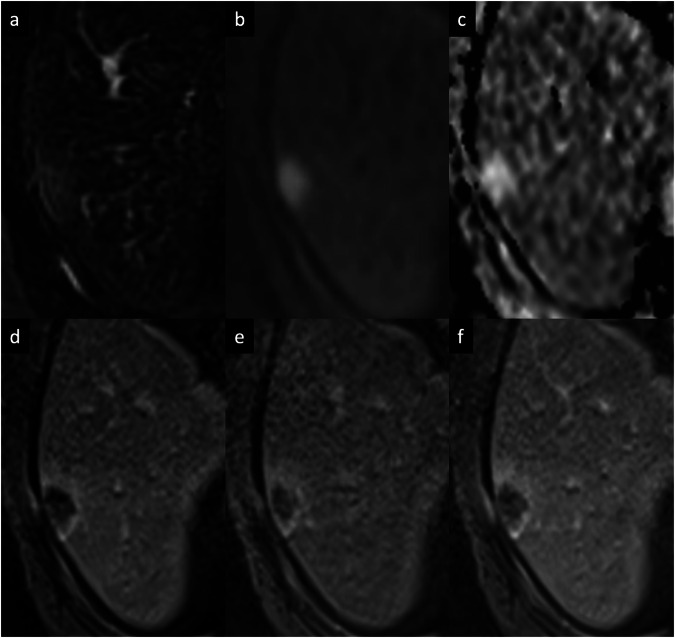
Solitary necrotic nodule in a 52-year-old man. Extracellular contrast agent-enhanced MRI shows a subcapsular lesion in the right hepatic lobe, (**a**) slightly hyperintense on T2-weighted images, (**b**) high SI on DWI, and (**c**) with high values on ADC map. **d** The lesion demonstrates a rim APHE that persisted on portal venous (**e**) and delayed (**f**) phases. A biopsy of the lesion confirmed the diagnosis of a solitary necrotic nodule in the context of homogenous and moderate hepatic steatosis

A solitary necrotic nodule of the liver is a rare benign lesion that might result from previous trauma, sequelae of previous parasite infection, or sclerosed hemangiomas [22]. This entity usually appears as a small solitary nodule, mainly found adjacent to the liver capsule of the right lobe. Imaging findings may depend on the natural history of the lesion [23]. Early in their development, the lesions may show hypoattenuation on unenhanced CT, low SI on T1-WI, hypo-to-isointense on T2-WI surrounded by a hyperintense halo, with rim APHE, and thin delayed rim enhancement (Fig. 5). At a later stage, key findings include reduced size, calcifications, low SI on both T1-WI and T2-WI, the complete lack of enhancement (due to intralesional necrosis), and variable amounts of intralesional hemorrhage [22, 23]. The differential diagnosis includes solitary metastasis and might require a percutaneous biopsy for confirmation [23].

### Vascular

#### Epithelioid hemangioendothelioma (EHE)

**Fig. 6:**
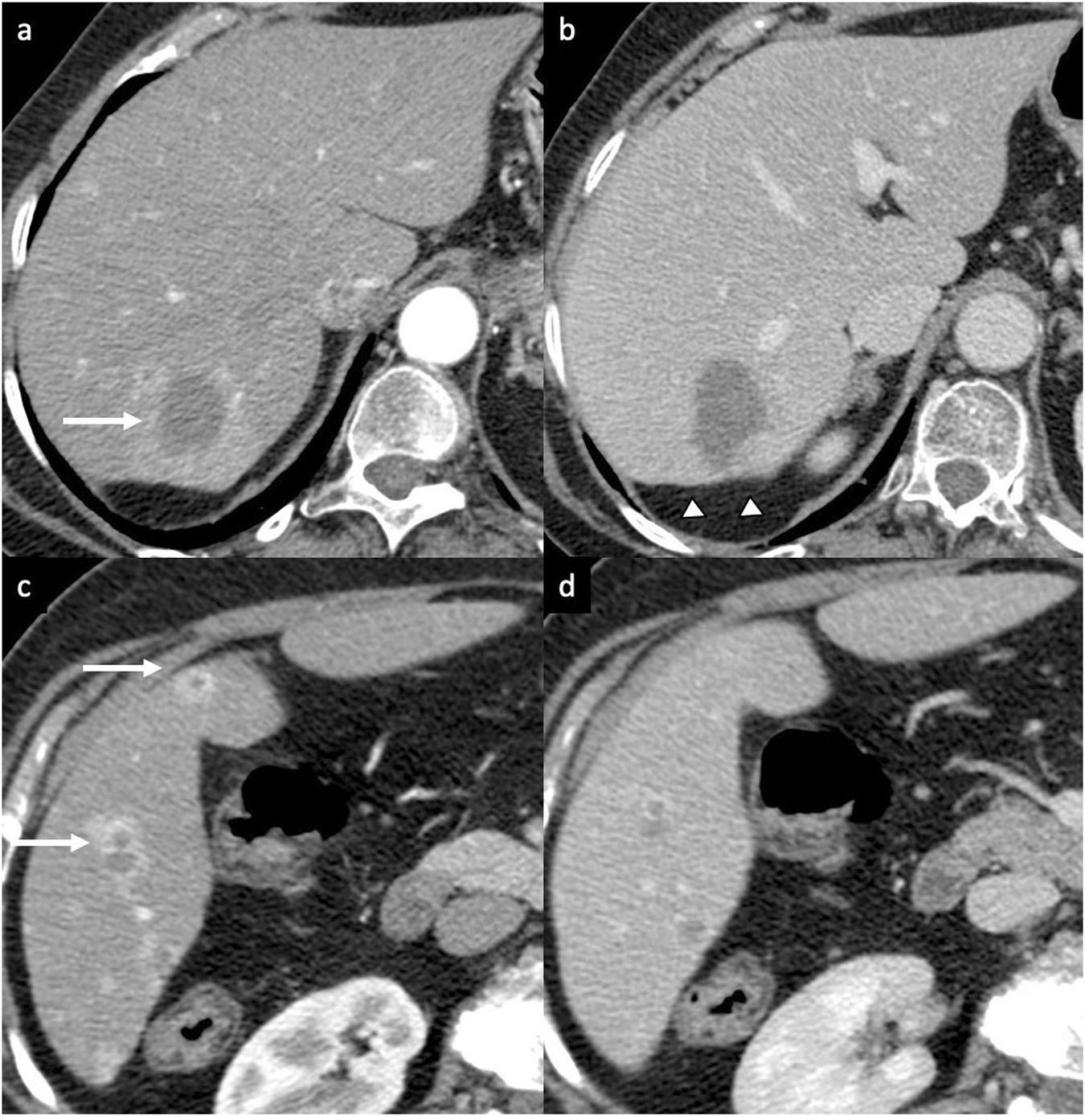
EHE in a 66-year-old woman. **a**, **c** Contrast-enhanced CT shows multiple liver lesions with nodular or irregular rim APHE (arrows) with (**b**, **d**) gradual enhancement on the portal venous phase. The lesions predominate in the peripheral regions of the liver (subcapsular). Note, the minimal retraction of the liver capsule (**b**, arrowheads). A biopsy of the liver confirmed the diagnosis of EHE

EHE is a rare vascular malignancy of mesenchymal origin [24]. The etiology is unknown; however, possible etiologic factors have been suggested, such as exposure to vinyl chloride, occupational contaminants, major trauma to the liver, and viral hepatitis [25]. Patients often have nonspecific symptoms; one-third of them have extrahepatic lesions at the initial diagnosis [24–26]. Tumor marker levels are usually within normal limits [24–26]. Typical imaging appearance includes multiple hypoattenuating nodules on unenhanced CT, ranging from 0.5 cm to 10 cm in diameter, that frequently coalesce and form larger confluent masses, with a propensity to involve the peripheral regions of the liver and to extend to the liver margin. Nonspecific findings, such as retraction of the liver capsule and intralesional calcifications, may be present [24–26]. Contrast-enhanced dynamic imaging shows nodular or irregular rim APHE followed by progressive enhancement of the central fibrous stroma on PVP and DP (“black target sign”) in 86.7% of cases [27] (Fig. 6). Some lesions are surrounded by a thin, non-enhancing hypodense rim caused by tumor invasion of hepatic sinusoids, venules, and small portal vein branches. On MRI, EHE shows heterogeneous low SI on T1-WI, moderately hyperintense peripheral rim and a markedly hyperintense central area on T2-WI, and a peripheral rim of high SI on DWI (“targetoid appearance”) [24–26]. Central areas of reduced SI may correspond to hemorrhage, coagulation necrosis, and calcifications [24–26]. Because EHE has the tendency to spread within the portal and hepatic vein branches, another specific finding is the “lollipop sign”, a combination of the well-defined tumor mass on enhanced images (the candy in the lollipop) and the adjacent occluded vein (the stick) [28]. Those signs are specific findings of HEH but they can also be seen in other entities, such as iCCA, abscesses, and liver metastases from various primary cancers (i.e., breast and colon cancer). In this context, key differentiating features are the peripheral location of the nodules, the capsular retraction, and the tendency to show coalescent multiple lesions. The definitive diagnosis requires histopathologic confirmation [25].

## Malignant

### iCCA

**Fig. 7:**
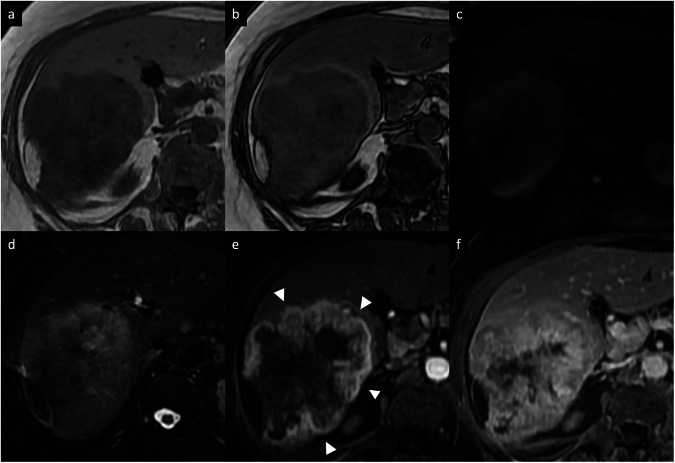
Intrahepatic mass-forming cholangiocarcinoma in a 62-year-old woman. **a** Axial T1-weighted in-phase and (**b**) opposed-phase MR images show a large lobulated hypointense mass, (**c**) with high SI on DWI (*b* = 800), (**d**) slightly hyperintense on fat-suppressed T2-weighted images, with associated capsular retraction. **e** Extracellular contrast agent-enhanced MRI sequences demonstrate a thick irregular rim APHE (arrowheads), (**f**) with progressive central enhancement on DP

iCCA is the most common primary non-HCC malignancy in the liver and it manifests with different morphological types and growth patterns [29]. The mass-forming type is the most common form of iCCA [29]. It classically manifests as a large lesion with irregular lobulated margins, rim APHE, progressive centripetal enhancement, and peripheral washout on PVP and DP [29, 30]. This enhancement pattern reflects the histology of the tumor, with viable tumor cells usually located at the periphery, with a central portion composed of a desmoplastic and hypovascularized tumor stroma with fibrosis and coagulative necrosis [31–33]. Other common imaging findings include capsular retraction, dilatation and thickening of the intrahepatic bile ducts around the tumor, vascular encasement by the tumor (but intravascular tumor invasion is rare), satellite nodules, intrahepatic metastases, and obliteration of the portal vein [33, 34]. On MRI, iCCA shows low-to-moderate SI on T2-WI and low SI on T1-WI [31–33]. iCCA may show the “necrosis imaging sign” as a persistent, nonenhancing defect with either high SI or low SI on the T2-WI. DWI demonstrates a target appearance on high *b*-value images (a central darker area due to fibrosis with peripheral hyperintense area) associated with peripheral hypointensity and central hyperintensity on the ADC map [31–34]. This “targetoid appearance” is also seen on the HBP of Gd-EOB-MRI, which indicates peripheral rim hypointensity and central cloud-like hyperintensity due to retained contrast material in the fibrotic stroma (“EOB-cloud enhancement”) (Fig. 7) [31–35]. Table 1 summarizes the main imaging features to differentiate mass-forming iCCA from its potential mimickers. Approximately 80% of scirrhous HCCs also showed the targetoid appearance on HBP [36], and the presence of T2 central darkness, a capsule, and septa on MRI are statistically significant features of scirrhous HCCs in comparison with ICCs [37]. The mucinous subtype of iCCA may show marked hyperintensity on T2-WI and centripetal enhancement pattern, but it should be distinguished from a hemangioma based on its continuous ragged peripheral enhancement, as opposed to the discontinuous nodular enhancement of the latter [38]. In iCCA, a rim APHE is the most frequently observed and sensitive LR-M feature (56.5–82.8%), followed by targetoid HBP on Gd-EOB-MRI (25.9–43.5%) and delayed central enhancement (24.2%) [39–41]. In assessing patients with LR-M lesions, serum tumor markers such as CA 19-9 may help diagnose iCCA [42]. The final diagnosis of LR-M observations requires histopathologic confirmation before treatment [39–42].

**Table 1:** Main differential diagnoses for mass-forming iCCA and their typical imaging features

Observations	Typical imaging features on CT and MRI
Hepatic abscess	Commonly in patients with intrahepatic stone disease Thick enhancing wall with central cystic change
Metastases	Central necrotic areas hyperintense on T2-WI and hypointense on T1-WI, with delayed contrast material uptake on the HBP
Sclerosing/fibrolamellar HCCs	Typical low SI on HBP Central calcification in FLC
cHCC-CCA	Tumoral vascular thrombosis Absence of intrahepatic bile duct dilatation Absence of the target appearance on HBP

*iCCA* intrahepatic cholangiocarcinoma, *HBP* hepatobiliary phase, *FLC* fibrolamellar hepatocellular carcinoma

### HCC

**Fig. 8:**
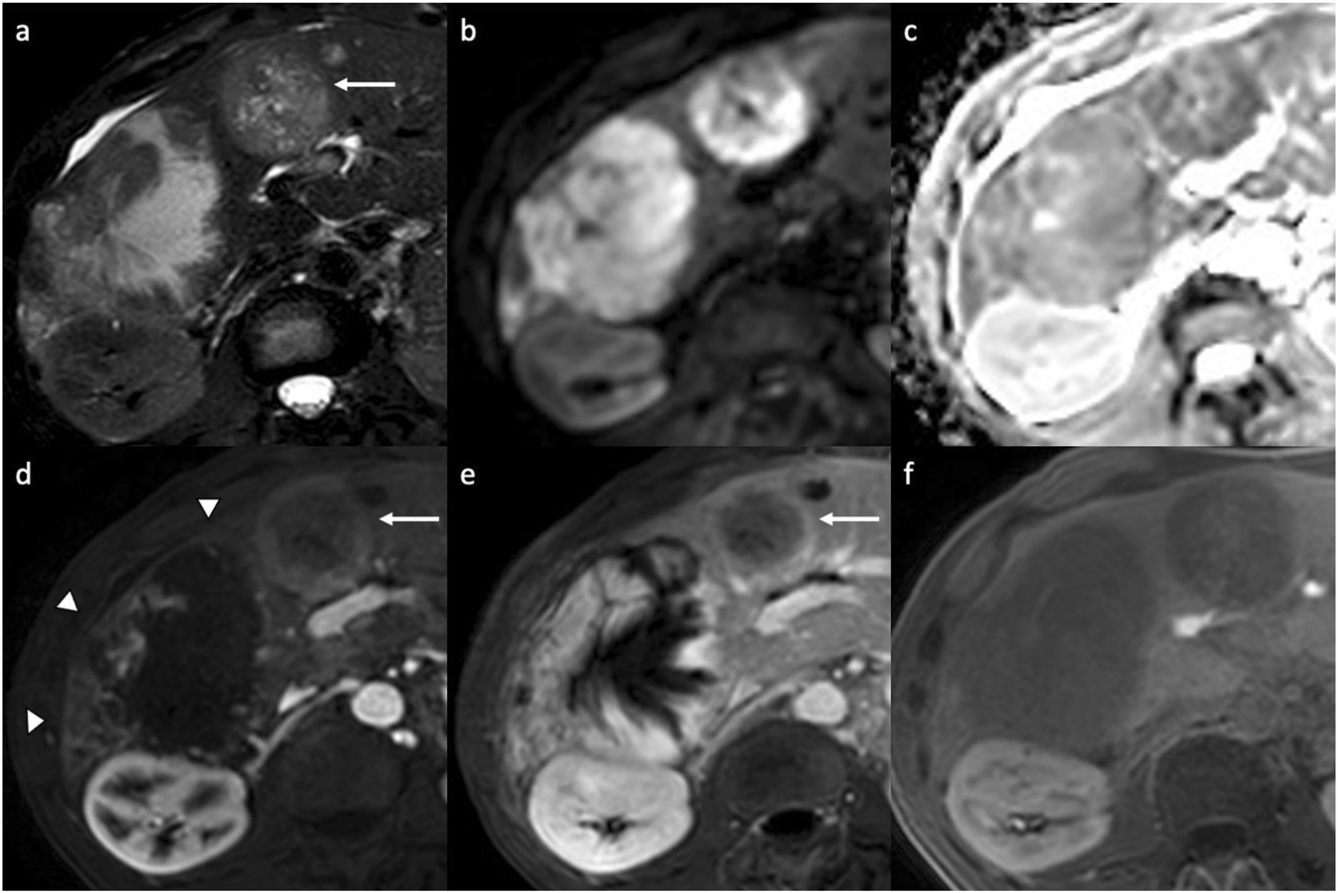
HCC and cavernous hemangioma in a 45-year-old man. **a** Axial T2-weighted MRI shows a subcapsular lesion with slightly high SI (arrow), with high SI on (**b**) DWI and low values on (**c**) the ADC map. **d** On gadobenate dimeglumine-enhanced MR sequences, the lesion shows a rim arterial phase enhancement (arrow), (**e**) capsule appearance in the delayed phase, and (**f**) low SI in the hepatobiliary phase, suggestive of HCC. Contrast-enhanced MRI also shows a large lobulated mass located in the right hepatic lobe, with high SI on (**a**) T2-weighted images, (**b**, **c**) without diffusion restriction, and with peripheral discontinuous nodular enhancement on (**d**) AP (arrowheads), followed by progressive centripetal enhancement on (**e**) DP (cavernous hemangioma)

HCC is the leading cause of cancer-related mortality in patients with chronic liver disease [43]. Approximately 90% of HCCs are associated with a known underlying etiology, most frequently chronic viral hepatitis (B and C), metabolic dysfunction-associated steatotic liver disease, and alcohol intake [43]. There are widely varying appearances of HCC on imaging. In noncirrhotic patients, HCC usually manifests as a large solitary mass (> 4 cm) that shows necrosis and central scar formation more frequently than in HCC developed in cirrhotic patients [44]. On dynamic CT and MRI, HCC typically shows the combination of nonrim APHE and nonperipheral washout on PVP or DP. The LI-RADS system also integrates the use of other imaging features, such as the presence of tumor-enhancing capsule, size, and significant tumor growth over time (> 50% in 6 months or less) [2, 45, 46]. However, some HCCs displaying an atypical enhancement pattern of peripheral rim APHE were reported in 5.6–15.7% of cases (HCCs with fibrotic components, poorly differentiated HCCs, sarcomatoid and scirrhous/sclerosing subtypes, HCCs with vessel encapsulating tumor clusters, or HCCs displaying progenitor cell markers) [47–50] (Fig. 8). The status of the patient (i.e., LI-RADS high risk of HCC or not) and the presence of other imaging ancillary features that favor the diagnosis of HCC, such as nonenhancing capsule, mosaic architecture, nodule-in-nodule architecture, intralesional fat, intralesional hemorrhage may guide the radiologists towards a diagnosis of HCC [2, 45, 51].

### Fibrolamellar hepatocellular carcinoma (FLC)

**Fig. 9:**
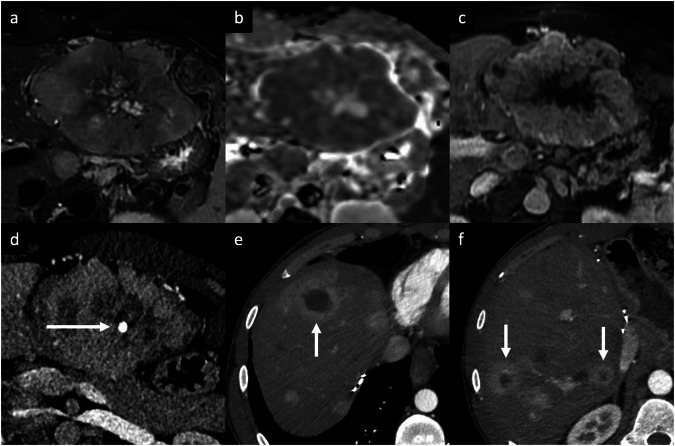
FLC in a 29-year-old woman. **a** Axial T2-weighted MR sequences show a mildly hyperintense heterogeneous mass in the left liver lobe, containing a T2-hypointense central scar, (**b**) with a targetoid appearance on an ADC map. **c** Extracellular contrast-enhanced AP image shows heterogeneous enhancement within the mass. **d** Axial contrast-enhanced AP CT demonstrates heterogeneous enhancement with typical central calcification (arrow). **e**, **f** Contrast-enhanced CT imaging performed after two years of a surgical tumor resection shows multiple tumor recurrences on the right hepatic lobe with rim APHE (arrows)

FLC is a very rare form of primary hepatic cancer, accounting for approximately 1% of all HCCs [52]. This tumor subtype occurs in young adults (second or third decade of life) without underlying hepatitis or cirrhosis (95% of cases) [53, 54]. FLC shows unique molecular oncogenic abnormalities with *DNAJB1*–*PRKACA* translocations. Patients frequently present with abdominal pain, malaise, weight loss, or a palpable abdominal mass or hepatomegaly; liver function tests may be normal or mildly elevated, and serum alpha-fetoprotein (AFP) is useless as a tumor marker [52–54]. Fibrolamellar HCC appears as a solitary, well-defined, large heterogeneous lesion with a lobulated surface and frequent central calcification [55]. On dynamic contrast-enhanced scans, FLC shows low attenuation compared with the surrounding liver, with thick rim APHE and variable enhancement pattern in PVP and DP; the fibrous central area and radial septa usually show delayed enhancement [55, 56]. Nodal metastases are common and occur in up to 50–65% of cases, most commonly seen at the hepatic hilum and hepatoduodenal ligament [57]. On MRI, the tumor is usually hypointense on T1-WI and hyperintense on T2-WI, with a central fibrous area that shows low SI on both T1-WI and T2-WI as well as low SI on the HBP of Gd-EOB-MRI (Fig. 9) [55–57]. The most common differential diagnosis is focal nodular hyperplasia, but the imaging features can overlap with those of other hyperenhancing lesions with central areas, including hepatocellular adenoma, hemangioma, metastases, and iCCA (Table 2). A biopsy may be required if there is any doubt in the diagnosis [55–57].

**Table 2:** Main differential diagnoses for FLC and their typical clinical and imaging features on contrast-enhanced imaging

Observations	Typical imaging features on CT and MRI
Focal nodular hyperplasia	Homogeneity Attenuation or SI similar to that of the surrounding liver Strong enhancement at AP without washout Central scar hyperintense on T2-WI Absence of capsule (lobulated aspect) Central calcification rare Different patterns of hyperintensity on HBP
Hemangioma	Marked hyperintensity on T2-WI Hyperintense on high *b*-values DWI, hyperintense on ADC map Peripheral discontinuous nodular APHE and gradual centripetal filling during PVP and DP
Hepatic adenoma	Homogeneous APHE (heterogeneous APHE in FLC) Isoattenuating or isointense to the liver on DPs
HCC	Nonrim APHE and washout on PVP or DP Intralesional fat

*HBP* hepatobiliary phase, *DWI* diffusion-weighted imaging, *ADC* apparent diffusion coefficient, *APHE* arterial phase hyperenhancement, *PVP* portal venous phase, *DP* delayed phase

### Combined hepatocellular-cholangiocarcinoma (cHCC-CCA) tumor

**Fig. 10:**
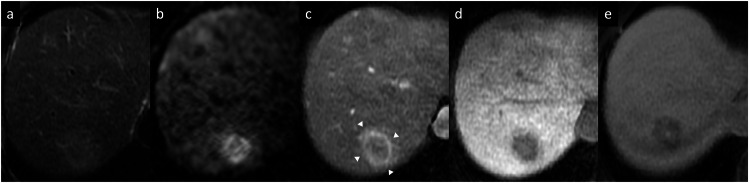
cHCC-CCA tumor in a 55-year-old man with hepatitis C-related cirrhosis. **a** Gadoxetate disodium magnetic MRI shows a heterogeneous hyperintense lesion on fat-suppressed T2-weighted images, (**b**) high SI on DWI, (**c**) contrast-enhanced T1-weighted images demonstrate an irregular rim APHE (arrowheads), and (**d**) low SI on hepatobiliary phase acquired 20 min after administration of hepatobiliary contrast agent. **e** On the hepatobiliary phase 2 h after administration of gadobenate dimeglumine, the periphery of the lesion is hypointense, while the central fibrotic areas show high SI (“targetoid appearance”). A biopsy of the liver confirmed the diagnosis of cHCC-CCA

cHCC-CCA is a rare primary liver cancer composed of elements from both histological entities, with a reported incidence of less than 1% among all primary liver cancers [58]. cHCC-CCA mainly develops in patients with chronic liver disease or cirrhosis [58, 59]. Laboratory findings include possible elevated levels of AFP and CA 19-9. The characteristics of cHCC-CCA depend on the proportions of tumor components, showing a mixture of both HCC and iCCA imaging features. On contrast-enhanced CT, cHCC-CCA appears as a hypoattenuating or isoattenuating lesion, with a variable pattern of enhancement: early peripheral rim APHE with central hyperenhancement and peripheral washout on the DP (concentric zones of HCC peripherally and CCA centrally), diffuse early APHE and washout and capsule on DP (classical hallmarks of HCC observed in a minority of cases) (Fig. 10) [60, 61]. On MRI, cHCC-CCA shows low SI on T1-WI, heterogeneous hyperintensity on T2-WI with or without central hypointense focus (central CCA or fibrotic component), and diffusion restriction on DWI. Targetoid appearance on the HBP of Gd-EOB-MRI, capsular retraction, bile duct dilatation, and lymph nodes are more suggestive of CCA-like lesions; venous invasion is typical of HCC-like lesions [60, 61]. According to the LI-RADSv2018, targetoid appearance suggests non-HCC malignancy but does not exclude HCC, and cHCC-CCA should be categorized as LR-M [2]. The association of HCC features with CCA features (appearance of iCCA with portal venous invasion, or appearance of HCC with biliary dilation or enlarged lymph nodes) may guide the diagnosis. Furthermore, in a liver without underlying disease, the differential diagnosis would include hepatocellular adenoma, FNH, and hyperenhancing metastasis. The combined interpretation of imaging features and biopsy offers better diagnostic performance of cHCC‐CCA and may be helpful to narrow differential diagnosis [62].

### Primary hepatic lymphoma (PHL)

PHL is a rare form of lymphoproliferative disorder confined to the liver and perihepatic nodal sites without distant lymphomatous involvement at patient presentation [63]. PHL is commonly associated with viral hepatitis B and C and Epstein–Barr virus, and most patients present with right upper quadrant pain or jaundice, while fever and weight loss are found in about one-third of patients [63]. PHL may manifest at imaging as a solitary focal liver lesion, multiple lesions, a diffuse infiltration, or an ill-defined mass in the porta hepatis [63, 64]. The most common imaging manifestation is a heterogeneous solitary lesion with soft-tissue attenuation, poorly enhancing, or a rim APHE [63, 64]. The lesions typically show vascular or biliary encasement without thrombosis or ductal and vessel dilatation/distortion (“vessel-penetrating sign”) [63, 64]. On MRI, the nodules tend to be hypo- or isointense on T1-WI, moderately hyperintense on T2-WI, or may show a “target appearance”, markedly restricted diffusion on DWI, and with low SI on HBP [65]. PHL has a wide range of differential diagnoses and can mimic many conditions, such as iCCA, HCC, inflammatory pseudotumor, primary hepatic neuroendocrine tumor, liver infections, and metastases. A definitive diagnosis by imaging remains a challenge, and a definitive diagnosis can often be achieved only through histopathologic examination.

### Metastases

**Fig. 11:**
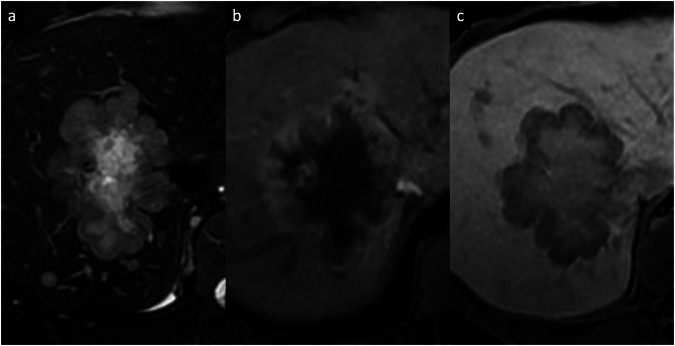
Synchronous liver metastasis from invasive ductal carcinoma of the breast in a 47-year-old woman. **a** Axial fat-suppressed T2-weighted MRI shows a large focal liver lesion with a central fibrotic component, depicted as an area of hyperintensity, whereas peripheral viable tumor is depicted as an area of moderate hyperintensity; this contrast between the peripheral and central areas is described as the so-called “target sign”. **b** Gadoxetate disodium-enhanced MRI demonstrates a rim APHE surrounding a central hypointense area. **c** The hepatobiliary phase shows a “cloud-like” appearance with a central portion that is relatively hyperintense compared to the hypointense peripheral area (“targetoid appearance”). A biopsy of the lesions confirmed the diagnosis of liver metastases from breast cancer

The liver is one of the most common sites of metastases, and liver metastases are more common than primary liver cancers [66]. Liver metastases are broadly classified as hypoenhancing and hyperenhancing relative to the liver parenchyma in the AP [66]. Among hypoenhancing metastases, adenocarcinoma from the gastrointestinal tract (colorectum, stomach, pancreas, and biliary system) is the most frequent source, while hyperenhancing metastases typically originate from neuroendocrine tumors, renal cell carcinoma, thyroid carcinoma, choriocarcinoma, pheochromocytoma, or soft-tissue sarcomas [66–68]. A large number of other malignant tumors from almost any site, e.g., gastrointestinal stromal tumor, malignant melanoma, and lymphoma, can metastasize to the liver [66–68]. In diagnosing liver metastases, several characteristic imaging findings need to be considered, such as tumor vascularity, attenuation values and signal intensities, and growth patterns. On contrast-enhanced CT and MRI, rim APHE has been recognized as one of the characteristic findings of hepatic metastases [67–70]. Hypoenhancing metastases tend to show an early appearance of rim APHE, while hyperenhancing ones show more delayed rim enhancement. On MRI, the use of hepatobiliary contrast agents, especially the combined image analysis using the Gd-EOB-MRI with HBP and DWI, yields better diagnostic performance and offers a higher sensitivity for detecting small liver metastases compared to contrast-enhanced CT or extracellular agents MRI [67–70]. Metastases are hypointense on HBP due to their lack of functional hepatocytes. However, metastases may occasionally demonstrate central areas of relative hyperintensity on HBP (described as “EOB-cloud enhancement” similar to cholangiocarcinoma) compared to surrounding lesion hypointensity, resulting in a target appearance (peripheral hypointense rim compared to central cloud of enhancement) [70, 71]; this finding represents a paradoxical uptake of gadoxetic acid in the central area of lesion owing to accumulation of contrast in fibrotic tissue, such in cases of metastases from colorectal and breast cancers [71]. Recognizing the characteristic imaging features of different liver metastases from various primary malignancies, considering also the patient history and the need for liver biopsy if no primary tumor is known with immunohistochemistry, is essential because treatment strategies can differ according to the primary tumor (Table 3) (Fig. 11).

**Table 3:** Liver metastases and their typical imaging features on contrast-enhanced CT and MRI

Observations	Typical imaging features on CT and MRI
Colorectal adenocarcinoma	Hypoenhancing lesions Peripheral APHE with peripheral washout on PVP and DP Target sign on T2-WI and HBP (central necrosis) Cystic appearance on T2-WI (mucinous subtype) Peritumoral hyperintensity on HBP Capsular retraction Calcifications may be present (mucinous subtype)
Gastric adenocarcinoma	Peripheral APHE with peripheral washout on PVP and DP Capsular retraction Calcifications may be present
Neuroendocrine tumors	Cystic appearance on T2-WI (severe necrosis or degeneration) Peripheral washout Intra-tumoral hemorrhage (fluid–fluid level) Peritumoral hyperintensity on HBP Hyperenhancing metastases
Gastrointestinal stromal tumor	Cystic appearance on T2-WI (necrosis or degeneration) Intra-tumoral hemorrhage Peritumoral hyperintensity on HBP
Breast adenocarcinoma	Target sign on T2-WI an HBP Peripheral APHE with peripheral washout on PVP and DP Capsular retraction Calcifications may be present
Testicular carcinoma	Cystic appearance on T2-WI (necrosis or degeneration) Intra-tumoral hemorrhage
Ovarian carcinoma, endometrial carcinoma	Cystic appearance on T2-WI (cystic components of primary tumor) Intra-tumoral hemorrhage (fluid–fluid level) Calcifications may be present

Target sign on T2-WI: the contrast between central (fibrous/hemorrhagic areas hypointense on T2-WI or liquefactive necrosis hyperintense on T2-WI) and peripheral areas (viable tumor with moderate hyperintensity on T2-WI)

Peripheral rim washout sign: enhancing lesion with a peripheral rim of decreased enhancement relative to its center and the surrounding parenchyma on DP MRI using extracellular contrast agents

Target sign on HBP: central hyperintense areas with lower Si compared with the background parenchyma

Peritumor hyperintensity on HBP: a homogeneously hyperintense rim surrounds the tumor

Cystic appearance on T2-WI: cystic changes due to necrosis or degeneration, cystic metastases, or marked hyperintensity on T2-WI due to the primary histologic features

Marked hyperintensity on T1-WI: presence of paramagnetic substances, such as melanin (melanoma), extracellular methemoglobin (hemorrhage), protein (ovarian adenocarcinoma, multiple myeloma, pancreatic mucinous cystic tumors), and necrosis (colorectal adenocarcinoma)

## Post-treatment viable tumor and non-tumoral changes

**Fig. 12:**
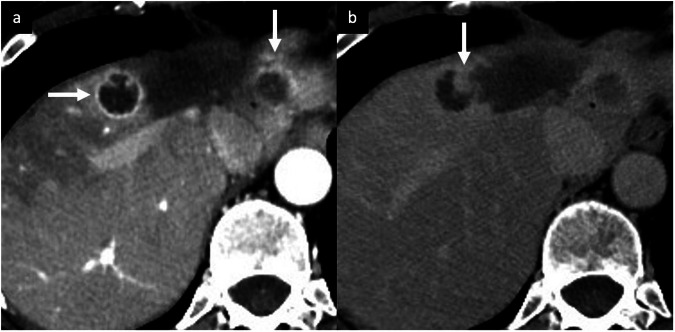
Multinodular tumor recurrence of iCCA in a 49-year-old woman. CT performed after local-regional treatment (transarterial radioembolization) demonstrates multinodular tumor recurrence around the treated zone showing irregular, thickened, nodular rim APHE (**a**, arrows) which persisted and increased (**b**) on DP (arrow)

After different locoregional treatments, such as radiofrequency ablation, microwave ablation, transarterial chemoembolization, and transarterial radioembolization, patients undergo multiphasic imaging to assess treatment response and to identify potential sites of progressive tumors elsewhere in the liver [72]. Recurrent or residual tumors may have a variety of imaging appearances. A complete lack of internal enhancement in treated tumors indicating complete tumor necrosis (rarely present at immediate postprocedural imaging) is classified as “LR-TR Nonviable” by the LI-RADS treatment response algorithm [72]. The presence of peripheral nodular or irregular rim enhancement should be classified as “LR-TR Viable” [2]. A well-described normal post-treatment finding is a smooth, thin, continuous rim APHE surrounding the treated zone without washout, usually corresponding to inflammation [72, 73]. This finding usually disappears within one month but may persist longer. Geographic APHE in the hepatic parenchyma adjacent or peripheral to the treatment zone is a normal finding that usually disappears 3–6 months after ablation [72, 73]. The key imaging features that suggest residual or recurrent tumor are an irregular, thickened, nodular, or mass-like APHE within or around the treated zone or the disruption of a smooth continuous peripheral rim enhancement, especially if it is not decreasing in size over time (Fig. 12) [72, 73]. Additionally, lack of washout and absence of mass-like T2-WI or DWI signal abnormality are helpful in differentiating benign post-treatment parenchymal enhancement from recurrent disease. At initial imaging after transarterial chemoembolization treatment, a completely treated tumor will usually be similar in size to the tumor at pretreatment imaging, and it becomes immediately nonenhancing, similar to thermal ablation [72, 73]. Postprocedural hemorrhage, inflammation, and liquefactive necrosis can also be present in the treatment zone and may result in a temporary increase in the size of the treated tumor [72, 73]. Similar to thermal ablation, there is commonly an inflammatory thin, continuous, smooth rim APHE surrounding an effectively treated tumor that may persist for more than one year; any associated thickening or nodularity should raise suspicion for viable tumor [72, 73]. Prior studies have suggested that radiomics analysis based on PVP and HBP of gadoxetate disodium-enhanced MRI may also be helpful in predicting response in HCCs treated with TAE [74]. Unlike ablation and transarterial chemoembolization, tumor necrosis after transarterial radioembolization is not immediate, and a persistent intratumoral enhancement (diffuse or nodular, central or peripheral) with or without washout or capsule may be seen in the first few months after treatment, even if the mass is completely treated [72, 73, 75, 76]. Peritumoral thin ring APHE without asymmetry, nodular morphology is a benign finding related to inflammation or parenchymal fibrosis that may persist for months after treatment [72, 73, 75, 76].

In conclusion, a broad spectrum of focal liver lesions may show rim APHE on dynamic imaging as a typical or an atypical presentation. The nature of benign and malignant liver lesions with rim APHE is variable and includes vascular, infectious, inflammatory, biliary, hepatocellular, and secondary neoplastic origin. The differential diagnosis at imaging is based on clinical characteristics, laboratory tests, and imaging findings. Histopathological examination may be required in selected cases.

## Abbreviations

ADC: Apparent diffusion coefficient
AFP: Alpha-fetoprotein
AP: Arterial phase
APHE: Arterial phase hyperenhancement
cHCC-CCA: Combined hepatocellular-cholangiocarcinoma
DP: Delayed phase
DWI: Diffusion-weighted imaging
EHE: Epithelioid hemangioendothelioma
FLC: Fibrolamellar hepatocellular carcinoma
Gd-EOB-MRI: Gadoxetate disodium-enhanced magnetic resonance imaging
HCC: Hepatocellular carcinoma
iCCA: Intrahepatic cholangiocarcinoma
LI-RADSv2018: Liver Imaging Reporting And Data System version 2018
PHL: Primary hepatic lymphoma
PVP: Portal venous phase
WI: Weighted imaging
